# Prevalence and risk factors of bacterial enteric pathogens in men who have sex with men: A cross-sectional study at the UK's largest sexual health service

**DOI:** 10.1016/j.jinf.2022.10.033

**Published:** 2023-01

**Authors:** Holly D. Mitchell, Gary Whitlock, Jey Zdravkov, Jenny Olsson, Panida Silalang, Megan Bardsley, Paula B. Blomquist, Claire Jenkins, Nicholas R. Thomson, Nigel Field, Gwenda Hughes

**Affiliations:** a Centre for Molecular Epidemiology and Translational Research, Institute for Global Health, University College London, Mortimer Market Centre, Off Capper Street, London, WC1E 6JB, UK; b The National Institute for Health Research Health Protection Research Unit (NIHR HPRU) in Blood Borne and Sexually Transmitted Infections at University College London, UK; c 56 Dean Street, Chelsea and Westminster Hospital National Health Service Foundation Trust, London, W1D 6AQ, UK; d Imperial College School of Medicine, Imperial College London, South Kensington Campus, London, SW7 2AZ, UK; e Clinical and Public Health Group, UK Health Security Agency, 61 Colindale Avenue, London, NW9 5EQ, UK; f The National Institute for Health Research Health Protection Research Unit (NIHR HPRU) in Gastrointestinal Infections at University of Liverpool, UK; g Parasites and Microbes, Wellcome Trust Sanger Institute, Genome Campus, Hinxton, CB10 1SA, UK; h Department of Pathogen Molecular Biology, London School of Hygiene and Tropical Medicine, Keppel Street, London, WC1E 7HT, UK

**Keywords:** Bacterial infections, Drug resistance, bacterial, Cross-sectional studies, Prevalence, Asymptomatic infections, Sexual behavior, Sexually transmitted diseases

## Abstract

**Objectives:**

Outbreaks of bacterial enteric pathogens (BEPs) in men who have sex with men (MSM) associated with antimicrobial resistance are a public health concern. We investigated the prevalence and risk factors of BEPs in MSM to inform infection control.

**Methods:**

We conducted a cross-sectional study at a London sexual health clinic between 20/12/2017 and 06/02/2018. Residual rectal swabs from MSM attending for sexually transmitted infection (STI) testing were anonymously tested for a range of BEPs using real-time PCR. A sub-set of samples were tested for the *mphA* gene (a marker of azithromycin resistance). Results were linked to electronic health records.

**Results:**

BEPs were detected in 207 of 2116 participants, giving an overall prevalence of 9.8% (95% CI 8.5%-11.1%) ranging from 0.8% (0.4%-1.2%) for *Shigella* to 4.9% (4.0%-5.9%) for Enteroaggregative *E. coli*. MSM with BEPs were more likely to have a history of bacterial STIs (*p* = 0.010), to report more sexual partners (*p*<0.001), and among HIV-negative MSM, to report current HIV pre-exposure prophylaxis use (*p*<0.001). Gastrointestinal symptoms were rare (1.7%) and not associated with BEPs. 41.3% of MSM with BEPs and 14.1% of those without BEPs carried *mphA* (*p*<0.001). Among the former, this was associated with a history of bacterial STIs (51.5% vs 31.1%, *p* = 0.003).

**Conclusions:**

One in ten MSM had a BEP detected and most did not report symptoms. *MphA* carriage was common, particularly among those with BEPs. Bacterial STI treatment might contribute to selection of resistant gut organisms, emphasising the need for better antimicrobial stewardship.

## Highlights

• One in ten MSM had a bacterial enteric pathogen detected, and most had no symptoms.
• Detection of a bacterial enteric pathogen was associated with STI-risk behaviours.
• Detection of *mphA*, a marker of azithromycin resistance, was common.
• Among MSM with bacterial enteric pathogens, *mphA* was associated with a previous STI.

## Introduction

Bacterial enteric pathogens (BEPs) are transmitted faecal-orally and cause diarrhoea, nausea and abdominal pain. Transmission usually occurs through contact with an infected person or exposure to contaminated food, water or surfaces.^1^^,^^2^ BEPs can also be transmitted through the ingestion of faecal matter during or after sexual activity, through direct oral-anal contact (rimming), or indirectly through oral sex after anal sex, or via fingers or fomites.^3^ In the last two decades, there have been an increasing number of BEP outbreaks among men who have sex with men (MSM) globally including *Shigella* spp.,^4^, ^5^, ^6^, ^7^ *Campylobacter* spp.,^8^^,^^9^ and Shiga toxin-producing *Escherichia coli* (STEC).^10^ The epidemiological characteristics of these outbreaks have been similar, linked to specific sexual practices, recreational drug-use, and HIV infection.^4^^,^^10^

The emergence and spread of resistance to antimicrobials, including macrolides and fluoroquinolones, in some of these pathogens is concerning. Of note is the development of azithromycin resistance in *Shigella* spp. circulating within sexual networks of MSM. Genomic studies have described the presence of genotypic markers conferring resistance to azithromycin (for example *mphA*), which may be selected for through off-target effects from antibiotics prescribed for sexually transmitted infections (STIs).^11^^,^^12^

Our understanding of the epidemiology of BEPs in MSM has evolved from analyses of laboratory surveillance data, clinical case reports and information collected during outbreak management. These data largely focus on symptomatic individuals who present to healthcare and have stool samples collected for microbiological investigations. These individuals likely represent only a small fraction of infections in the population, including what might be a large burden of asymptomatic carriage. Although asymptomatic screening is an important public health tool for other STIs, routine screening for asymptomatic carriage of BEPs is not currently recommended, not least because the clinical implications and risks of onward transmission are poorly understood. Antimicrobial treatment is not clinically indicated unless the individual has severe symptoms or is at risk of complications (e.g. immunosuppression).^1^ Our previous UK-based feasibility study found that among 444 MSM diagnosed with rectal chlamydia at selected sexual health clinics (SHCs) in 2012, 8.6% (95% CI: 6.3% to 11.6%) had a BEP coinfection.^13^ About half of the cases in which a pathogen was detected did not report relevant symptoms suggesting that asymptomatic carriage might help to sustain BEP transmission among MSM. However, this small convenience sample had a known bias towards higher risk participants and lacked behavioural information.

We therefore conducted a large-scale cross-sectional study to estimate the prevalence of BEPs in an unselected sample of symptomatic and asymptomatic MSM attending a central London SHC for routine STI testing and care. We investigated the socio-demographic, clinical and behavioural risk factors of BEPs that might inform the design of interventions in this population. Given the potential importance of AMR, we also explored carriage of the *mphA* gene and its relationship to STI history.

## Methods

### Study design

We conducted this cross-sectional study at 56 Dean Street (56DS), the UK's largest sexual health and HIV service, between 20th December 2017 and 6th February 2018. We collected residual rectal swabs from consecutive adult men who attended the clinic and had a rectal swab taken for routine *Chlamydia trachomatis* and *Neisseria gonorrhoeae* testing. Swabs were sent to the Gastrointestinal Bacteria Reference Unit at the UK Health Security Agency (formerly Public Health England) and anonymously tested for selected BEPs. The results obtained from BEP detection were linked to clinical, socio-demographic and behavioural data extracted from the clinic database and the national GUMCAD STI Surveillance System.^14^

### Study population and specimen collection

56DS consists of two services, Dean Street Express (DSE) and 56 Dean Street (56DS). The former offers sexual health screening for asymptomatic people, and the latter offers testing and/or management for people with symptoms, those needing ongoing support or those requiring specialist services such as HIV post-exposure prophylaxis or HIV care.^15^^,^^16^ Approximately 75% of all *C. trachomatis* and *N. gonorrhoeae* tests performed by the service are carried out among those attending DSE.^17^ MSM attending the service are routinely offered testing for *C. trachomatis* and *N. gonorrhoeae* from urine, pharyngeal and rectal swabs, regardless of symptoms.

We collected residual rectal swabs from adult men aged 16 years and older, regardless of STI test results or symptom presentation. Although most rectal swabs are collected from men who identify as gay or bisexual, rectal swabs are also collected based on self-reported sexual behaviour and a smaller number are collected from individuals who identify as heterosexual or non-binary. All specimens were labelled with the participant's clinic number and attendance date only. Prior to testing, the clinic number was removed and replaced with an anonymous study number.

Assuming 5% BEP prevalence,^13^ 1825 rectal swabs were required to estimate prevalence to within 1% and detect a difference of 5% between two unequally sized sub-groups at 5% significance and >90% power, if sub-group prevalence was 9% and 4%. The target sample size was increased by 20% to 2281 as contingency for missing data items, failed DNA extractions or insufficient testing volumes.

### Data collection, linkage and anonymisation

Socio-demographic and clinical data were extracted from the GUMCAD STI Surveillance System using the participant's clinic number. GUMCAD is a pseudo-anonymised patient-level dataset that contains information about attendances, STI and HIV testing, and diagnoses made at all SHCs in England.^14^ Each record contains demographic information including gender, age, sexual orientation, ethnicity, country of birth and area of residence. Patient records can be linked longitudinally within but not across clinics using the participant's clinic number to determine HIV status and STI diagnosis history.

Additional clinical and behavioural data were extracted from the SHC database using the participant's clinic number. These data were collected locally as part of routine care using a standardised questionnaire completed using a touchscreen computer (DSE) or by the clinician during a face-to-face consultation (56DS). The questionnaire collected data on number of sexual partners (past three months), last condomless sex, current use of HIV pre-exposure prophylaxis (PrEP) and interest in specific ‘high-risk’ behavioural practices by asking, “Are you into any of these?: Fisting, injecting, bare backing, chemsex”. Hereon, this variable is referred to as ‘interest in specific high-risk practices’. We also extracted data on reported symptoms of gastrointestinal illness. While people attending DSE are not expected to have symptoms, some reported symptoms in the free text field. All people attending 56DS are routinely asked about symptoms, including gastrointestinal symptoms, which are recorded as free text.

All data were anonymised by replacing the participant's clinic number with an anonymous study number. Data were grouped into categories where appropriate to further minimise the risk of deductive participant identification (e.g. age).

### Laboratory procedures

DNA was extracted using the QIAsymphony DSP DNA Mini Kit (Qiagen). All specimens were spiked with 10 µl of modified green fluorescent protein *E. coli*,^18^ which acted as an internal positive control to minimise the risk of false negative reporting. Eluted DNA extracts were used to detect a range of BEPs using real-time polymerase chain reaction (PCR) primers and probes on a Rotor-Gene Q (Qiagen). The multiplex PCR assay included gene targets for *Shigella* spp./enteroinvasive *E. coli, Campylobacter jejuni/coli, Salmonella* spp., Shiga toxin-producing *E. coli* (STEC), enteroaggregative *E. coli* (EAEC), and enteropathogenic *E. coli* (EPEC). The amplification parameters were 95 °C for 5 min, followed by 95 °C for 15 s and 60 °C for 60 s (40 cycles). The cycle threshold was set at 0.05 for all targets. In a secondary analysis, real-time PCR using the Applied Biosystems TaqMan 7500 (Thermo Fisher Scientific) was used to detect the presence of *mphA*, an AMR gene associated with resistance to the macrolide, azithromycin.^19^ The PCR assay was performed using all eluted DNA extracts which returned a positive result for one of the BEP target genes, and for comparison, a random subset of 100 DNA extracts which returned a negative result for all BEP gene targets. Details of all the primers, probes and gene targets are provided in Supplementary Table 1.

### Statistical analyses

To assess representativeness of the study population, we compared their socio-demographic characteristics to those of all MSM attending 56DS or DSE, and all SHCs in England during the study period. Behavioural characteristics of the study population were compared with data available from an enhanced surveillance pilot at selected SHCs (2015 to 2016),^20^ and with data from a general population survey known as the third National Survey of Sexual Attitudes and Lifestyles (Natsal-3, 2010 to 2012).^21^

Prevalence estimates were calculated with 95% confidence intervals (CIs) using the Clopper-Pearson (exact binomial) method. The association between the detection of any BEP and clinical, socio-demographic and behavioural risk factors were explored using univariable and multivariable Poisson regression with robust error variances. For multivariable analyses, each exposure variable (i.e. socio-demographic, clinical or behavioural factor) was adjusted in a separate model *a priori* for age group, clinic (56DS or DSE) and HIV status. Overall p-values for heterogeneity were calculated using the Wald test and p-values for the test for linear trend were calculated for age group and number of sexual partners. Risk factor analyses stratified by HIV status were also conducted.

Sensitivity analyses using simple imputation methods were conducted to assess the potential bias of missing data (independent variables) on the results (Supplementary Data). Multiple imputation was not considered appropriate since descriptive analyses suggested the missing data mechanism was ‘missing not at random’.

The prevalence of *mphA* was calculated with 95% CIs using the Clopper-Pearson (exact binomial) method. The association between *mphA* detection and diagnosis of a bacterial STI in the past year was explored using Pearson's Chi-squared test.

### Ethical considerations

The study was reviewed and approved by the London Harrow NHS Research Ethics Committee (reference: 17/LO/1722). Individual patient consent was not required because we tested anonymised residual specimens with no patient-identifiable data. Posters and leaflets were displayed in the clinic waiting areas to inform patients about the study and they could opt out if they preferred to have their specimen excluded.

## Results

**Fig. 1:**
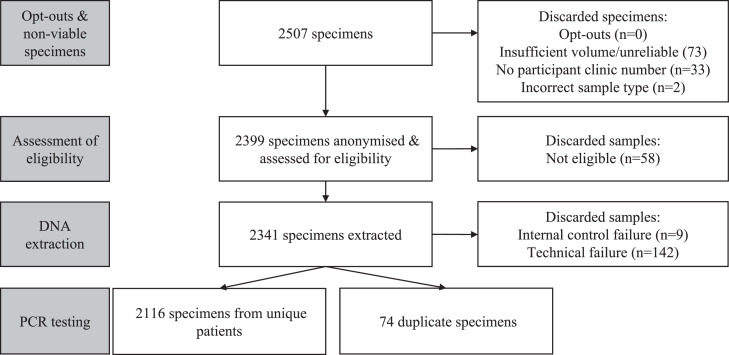
Flow chart showing the number of rectal swabs processed, assessed for eligibility, and tested.

Between 20/12/2017 and 06/02/2018, 2507 specimens were received, of which 2399 were assessed for eligibility (Fig. 1). 2190 eligible specimens generated a valid PCR test result, of which 2116 belonged to unique individuals and were included in the analysis. Linked GUMCAD data were available for 99.6% (2107) participants and additional clinical and behavioural data (from the clinic database) for 98.4% (2082).

Overall, 80.8% (1709) of study participants attended DSE and 19.2% (407) 56DS (Table 1). Most men reported gay identity (96.2%) and white ethnicity (77.8%) and nearly half were born in the UK (47.2%).

**Table 1:** Socio-demographic, clinical and behavioural characteristics of the study population.

Characteristic	Number	Percentage (%)
*Age group*		
16–19	31	1.5
20–24	241	11.4
25–29	526	25.0
30–34	485	23.0
35–39	337	16.0
40–49	339	16.1
50+	148	7.0
Missing	9	NA
*Ethnic group*		
White	1576	77.8
Black	76	3.8
Mixed	131	6.5
Asian	112	5.5
Other	130	6.4
Missing	91	NA
*World region of birth*		
UK	953	47.2
Europe (excluding UK)	581	28.8
Other	485	24.0
Missing	97	NA
*Sexual orientation*		
Gay	2003	96.2
Bisexual	54	2.6
Heterosexual	25	1.2
Missing	34	
*IMD quintile of residence*		
1–2 (Most deprived)	1407	67.5
3	374	17.9
4–5 (Least deprived)	304	14.6
Missing	31	NA
*Number of sexual partners (past 3 months)*		
0	12	0.7
1	170	10.0
2–4	678	39.8
5–9	440	25.9
10+	402	23.6
Missing	414	NA
*Number of new sexual partners (past 3 months)*		
0	172	10.6
1	270	16.7
2–4	588	36.3
5–9	347	21.4
10+	245	15.1
Missing	494	NA
*Receptive anal sex (past 3 months)*		
No	92	4.8
Yes	1816	95.2
Missing	208	NA
*Receptive oral sex (past 3 months)*		
No	47	2.5
Yes	1827	97.5
Missing	242	NA
*Last condomless sex*		
Never	184	9.8
More than 6 weeks ago	437	23.2
Within 6 weeks	986	52.4
Within 72 h	275	14.6
Missing	234	NA
*Interest in specific high-risk practices*^a^		
No	1074	60.5
Yes	701	39.5
Missing	341	NA
*Bacterial STI diagnosis (at attendance)*		
No/unknown	1632	77.1
Yes	484	22.9
*Bacterial STI diagnosis (past year)*		
No/unknown	1251	59.1
Yes	865	40.9
HIV status		
Negative/unknown	1744	82.4
Living with HIV	372	17.6
*Currently using HIV PrEP (*N* = 1744)*		
No	930	63.0
Yes	547	37.0
Missing	267	NA

*N* = 2116 unless otherwise specified. Missing data excluded from percentage calculations. Abbreviations: NA, Not applicable; IMD, Index of Multiple Deprivation; STI, Sexually Transmitted Infection; PrEP, Pre-exposure prophylaxis.

a ‘Interest in specific high-risk practices’ refers to data collected via the following question: Are you into any of these: Fisting, injecting, bare backing, chemsex.

The median number of total and new sexual partners in the past three months was four (IQR 2–9) and three (IQR 1–6), respectively. Overall, 17.6% (372/2116) of study participants were living with HIV. Among men who were HIV-negative or of unknown HIV status, 75.5% (1316/1739) tested HIV-negative at the study attendance and a further 23.7% (412/1739) within the past year. Where linked clinical data were available, 1.7% (36/2082) reported gastrointestinal symptoms.

The socio-demographic characteristics of the study population broadly mirrored those of all MSM attending the clinic during the study period, although a slightly higher proportion of study participants were of an ethnic minority background and born outside of the UK (Supplementary Table 2). Among men who reported at least one sexual partner in the past three months, 49.8% (842/1690) of study participants reported five or more sexual partners compared to 19.4% (164/844) of MSM attending clinics participating in the enhanced surveillance pilot, and 14% (17/123) of MSM in the general population in Natsal-3.

207 out of 2116 men had a BEP detected giving an estimated overall prevalence of 9.8% (95% CI: 8.5% to 11.1%). Prevalence was 0.8% (95% CI: 0.4% to 1.2%) for *Shigella* spp., 1.2% (95% CI: 0.8% to 1.8%) for STEC, 1.7% (95% CI: 1.2 to 2.3) for *Campylobacter* spp. and EPEC, and 4.9% (95% CI: 4.0% to 5.9%) for EAEC. *Salmonella* spp. were not detected.

There was no evidence of an association between BEP detection and socio-demographic factors (ethnic group, region of birth, Index of Multiple Deprivation quintile or sexual orientation), except that men of mixed ethnic background had a lower prevalence than white men (adjusted prevalence ratio [aPR]: 0.37 [95% CI: 0.16 to 0.89]), though the sample size for men of mixed ethnicity was small (*n* = 5/131) (Table 2).

**Table 2:** Associations of socio-demographic, clinical and behavioural factors with the detection of any bacterial enteric pathogen.

Factor	n/N	Row%	Unadjusted PR (95% CI)	p-value	Adjusted PR (95% CI)	p-value
*Clinic (*N* = 2116)*						
DSE	174/1709	10.2	1.00	0.210	1.00	0.139
56DS	33/407	8.1	0.80 (0.56–1.14)		0.76 (0.53–1.09)	
*Age group (*N* = 2107)*						
16–24	19/272	7.0	1.00	0.173	1.00	0.268
25–34	98/1011	9.7	1.39 (0.86–2.23)	0.068^a^	1.35 (0.84–2.17)	0.112^a^
35+	90/824	10.9	1.56 (0.97–2.52)		1.49 (0.92–2.43)	
*Ethnic group (*N* = 2025)*						
White	169/1576	10.7	1.00	0.161	1.00	0.198
Black	8/76	10.5	0.98 (0.50–1.92)		0.97 (0.50–1.90)	
Mixed	5/131	3.8	0.36 (0.15–0.85)		0.37 (0.16–0.89)	
Asian	10/112	8.9	0.83 (0.45–1.53)		0.88 (0.48–1.62)	
Other	10/130	7.7	0.72 (0.39–1.32)		0.72 (0.39–1.32)	
*Region of birth (*N* = 2019)*						
UK	90/953	9.4	1.00	0.546	1.00	0.575
Europe	65/581	11.2	1.18 (0.87–1.60)		1.17 (0.87–1.59)	
Rest of world	49/485	10.1	1.07 (0.77–1.49)		1.04 (0.75–1.45)	
*IMD quintile of residence (*N* = 2085)*						
1–2 (Most deprived)	144/1407	10.2	1.00	0.611	1.00	0.604
3	33/374	8.8	0.86 (0.60–1.24)		0.86 (0.60–1.23)	
4–5 (Least deprived)	27/304	8.9	0.87 (0.59–1.28)		0.87 (0.58–1.28)	
*Sexual orientation (*N* = 2082)*						
Gay	200/2003	10.0	1.00	0.298	1.00	0.357
Bisexual/heterosexual	5/79	6.3	0.63 (0.27–1.50)		0.67 (0.28–1.58)	
*HIV status (*N* = 2116)*						
HIV-negative/unknown	163/1744	9.4	1.00	0.141	1.00	0.198
Living with HIV	44/372	11.8	1.27 (0.92–1.73)		1.24 (0.89–1.73)	
*HIV risk group (*N* = 1849)*						
HIV-negative/unknown HIV status, not on HIV PrEP	60/930	6.5	1.00	<0.001	1.00	<0.001
HIV-negative, on HIV PrEP	74/547	13.5	2.10 (1.52–2.90)		2.06 (1.48–2.86)	
Living with HIV	44/372	11.8	1.83 (1.27–2.65)		1.85 (1.25–2.75)	
*Bacterial STI diagnosed at attendance (*N* = 2116)*						
No/unknown	145/1632	8.9	1.00	0.010	1.00	0.010
Yes	62/484	12.8	1.44 (1.09–1.91)		1.45 (1.09–1.91)	
*Bacterial STI diagnosed in past year (*N* = 2116)*						
No/unknown	103/1251	8.2	1.00	0.004	1.00	0.011
Yes	104/865	12.0	1.46 (1.13–1.89)		1.41 (1.08–1.84)	
*Interest in specific high-risk practices*^b^ (*N* = *1775*)						
No	97/1074	9.0	1.00	0.036	1.00	0.102
Yes	85/701	12.1	1.34 (1.02–1.77)		1.27 (0.95–1.69)	
*Number of sexual partners in last 3 months* (*N* = *1702*)						
0–1	14/182	7.7	1.00	0.005	1.00	0.010
2–4	52/678	7.7	1.00 (0.57–1.76)	<0.001^a^	0.97 (0.55–1.72)	0.003^a^
5–9	47/440	10.7	1.39 (0.78–2.46)		1.34 (0.75–2.37)	
10+	57/402	14.2	1.84 (1.06–3.22)		1.74 (1.00–3.05)	
*Number of new sexual partners in last 3 months* (*N* = *1622*)						
0–1	28/442	6.3	1.00	<0.001	1.00	<0.001
2–4	54/588	9.2	1.45 (0.93–2.25)	<0.001^a^	1.47 (0.95–2.26)	<0.001^a^
5–9	45/347	13.0	2.05 (1.30–3.21)		2.02 (1.29–3.16)	
10+	38/245	15.5	2.45 (1.54–3.89)		2.40 (1.51–3.80)	
*Receptive anal sex in last 3 months* (*N* = *1908*)						
No	7/92	7.6	1.00	0.475	1.00	0.547
Yes	180/1816	9.9	1.30 (0.63–2.69)		1.25 (0.61–2.54)	
*Receptive oral sex in last 3 months* (*N* = *1874*)						
No	6/47	12.8	1.00	0.486	1.00	0.410
Yes	178/1827	9.7	0.76 (0.36–1.63)		0.73 (0.35–1.54)	
*Last condomless sex* (*N* = *1882*)						
Never/more than 6 weeks ago	54/621	8.7	1.00	0.062	1.00	0.098
Within 6 weeks	99/986	10.0	1.15 (0.84–1.58)		1.11 (0.81–1.53)	
Within 72 h	38/275	13.8	1.59 (1.08–2.35)		1.52 (1.03–2.26)	
*Gastrointestinal symptoms* (*N* = *2082*)						
No/unknown	201/2046	9.8	1.00	0.410	1.00	0.184
Yes	5/36	13.9	1.41 (0.62–3.22)		1.78 (0.76–4.18)	

Total numbers vary for each question due to missing data. Unadjusted and adjusted prevalence ratios (PRs) and 95% confidence intervals (CIs) calculated using modified Poisson regression with robust error variance. Overall p-values by Wald test or linear test for trend.

Abbreviations: IMD, Index of Multiple Deprivation; STI, Sexually Transmitted Infection; PrEP, Pre-Exposure Prophylaxis.

a Adjusted Models: Each factor adjusted in a separate model for age group (linear term), clinic and HIV status.

b ‘Interest in specific high-risk practices’ refers to data collected via the following question: “Are you into any of these: Fisting, injecting, bare backing, chemsex”.

In univariable and multivariable analyses, BEP detection was positively associated with increasing numbers of total (linear test for trend *p* = 0.003) or new (linear test for trend *p*<0.001) sexual partners in the previous three months and a concurrent (aPR: 1.45 [95% CI: 1.09 to 1.91], *p* = 0.010) or previous (aPR: 1.41 [95% CI: 1.08 to 1.84] in previous 12 months, *p* = 0.011) bacterial STI diagnosis (Table 2). HIV status was not associated with BEP detection (aPR: 1.24 [95% CI: 0.89 to 1.73] for men living with HIV compared to men who were of HIV-negative/unknown HIV status, *p* = 0.198). However, after stratifying by current PrEP use (‘HIV risk group’ in Table 2), men without HIV and using PrEP and men living with HIV were more likely to have a BEP detected than men without HIV who were not taking PrEP (aPR: 2.06 [95% CI: 1.48 to 2.86] for men without HIV taking PrEP and aPR: 1.85 [95% CI: 1.25 to 2.75] for men living with HIV, *p*<0.001). Sensitivity analyses using simple imputation methods supported the findings presented in the primary analysis (Supplementary Data).

In both univariable and multivariable analyses stratifying by HIV status, BEP detection was strongly associated with STI-risk behaviours among MSM without HIV or of unknown HIV status but was not among MSM living with HIV (Supplementary Table 3; Supplementary Table 4; Supplementary Data).

**Fig. 2:**
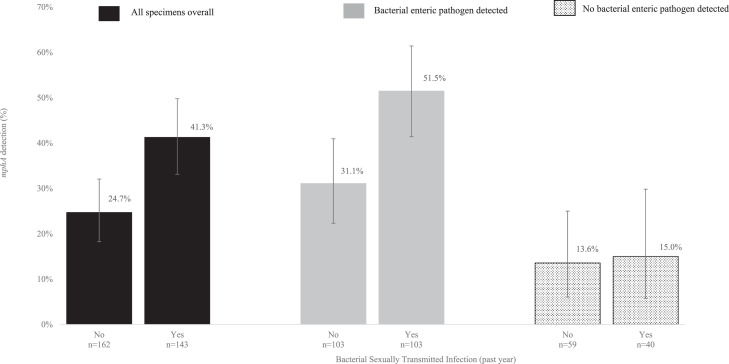
Detection of *mphA* by bacterial STI history in the past year stratified by bacterial enteric pathogen detection.

A total of 307 specimens (207 BEP-positive and 100 BEP-negative) were tested for the presence of *mphA.* One BEP-positive specimen had insufficient DNA volume to enable *mphA* testing, and one from the control group generated a negative result for the internal amplification control and was excluded. The *mphA* gene was detected in 32.5% (99/305) of specimens overall; detection was higher in BEP-positive specimens compared to the control group samples (41.3% [85/206] vs 14.1% [14/99], *p*<0.001). Overall, 41.3% (59/143) and 24.7% (40/162) of specimens from men with and without a bacterial STI in the past year, respectively, had *mphA* detected (*p* = 0.002) (Fig. 2). Among the sub-group with a BEP-positive specimen, *mphA* was detected in 51.5% (53/103) and 31.1% (32/103) of specimens from those with and without a bacterial STI diagnosis in the past year, respectively, (*p* = 0.003). Among the BEP-negative control group, *mphA* was detected in 15.0% (6/40) and 13.6% (8/59) of specimens from men with and without a bacterial STI in the past year, respectively (*p* = 0.840).

## Discussion

We found that one in ten men attending 56DS or DSE were PCR positive for a BEP with most reporting no clinical symptoms of gastroenteritis. BEP detection was associated with STI-risk behaviours, providing further strong evidence that these pathogens are transmitted through sexual contact. The *mphA* gene was most common in MSM with BEPs, but also common in those without, emphasising the frequency of carriage of resistance to the macrolide azithromycin in this population. Among those with BEPs, *mphA* was strongly associated with a bacterial STI diagnosis in the past year, likely reflecting a strong selective pressure through prior macrolide usage - azithromycin was used as part of first-line therapy for bacterial STIs prior to and during the study period.^22^

The main strength of this study was the large and unselected sample of MSM attending the UK's largest SHC, regardless of symptoms. The use of residual rectal swabs with an opt-out approach maximised the proportion of men attending the clinic who were included in the study and avoided selection bias. The study provides the most robust estimates of BEP prevalence among MSM in the UK to date. To our knowledge, this is also the first study to explore carriage of *mphA* in MSM, and its relationship with BEPs and bacterial STI diagnoses.

The main limitation was that our analyses used only routinely collected data due to the opt-out study design. For example, we could not collect additional information about gastrointestinal symptoms, antimicrobial exposure, travel history, occupational exposure, food consumption, participant knowledge of BEP transmission, or specific sexual practices facilitating faecal-oral transmission (such as direct oral-anal contact), all of which could have helped interpretation. Routinely collected data may be biased by systematic differences in data collection due to inaccurate recall or reporting, or missing responses. For some variables, there was a high proportion of missing data (Supplementary Data), potentially leading to biased estimates in risk factor analyses. However, sensitivity analyses assessing the impact of missing data were broadly concordant with the main results. Another limitation was that our PCR only detected the presence of the *mphA* gene, so we have likely underestimated off-target effects of antibiotic use in this population.

Our findings show that BEPs were present in the gastrointestinal tract of MSM attending 56DS and DSE, including pathogens associated with recent outbreaks globally. Although not directly comparable, our prevalence estimates were generally higher than in asymptomatic people included in a UK general population study,^23^ although this was conducted over 20 years ago and the incidence of enteric infections has since changed.^24^ Differences in study population characteristics, specimen type and testing methodology also hamper comparisons.^25^ A more recent general population cohort study among people who developed symptoms did not detect *Shigella* spp. and the prevalence of EAEC was considerably lower than in our study.^26^ In contrast, *Campylobacter* spp. were detected more frequently, which is consistent with this organism being the most common cause of gastrointestinal illness in the UK through the consumption of contaminated food.^27^ While the detection of *Salmonella* spp. was low in the general population cohort, this BEP was entirely absent in our study.

Overall, the association between BEP detection and STI-risk behaviours strengthens the evidence that transmission of these pathogens is an important public health concern for MSM. The association was less clear in MSM living with HIV, possibly because this group was generally more likely to report STI-risk behaviours compared to other MSM. However, caution is needed in interpretation due to the small sample size.

Our prevalence estimates are comparable to a study carried out among 519 asymptomatic (defined as no diarrhoea in the past two weeks) MSM attending a SHC in Melbourne, Australia during November 2018 and February 2019.^28^ In that study, prevalence of at least one bacterial, viral or protozoan enteric pathogen in rectal swabs was 11.0% (95% CI: 8.4% to 14.0%) and detection was independently associated with direct oral-anal contact in the past 12 months and group sex in the past month. In contrast to our study, there was no evidence that the detection of enteric pathogens differed by HIV or PrEP status.

As with other STIs, our findings suggest that partner change is an important component in the spread of BEPs in MSM. In turn, this suggests that MSM at highest risk of BEPs would benefit from targeted health education and risk reduction advice (for example, on the risks of sexual behaviours that facilitate transmission of enteric pathogens), while those diagnosed should be given appropriate advice to prevent transmission, and their partners notified. The absence of symptoms in most men strongly suggests that asymptomatic carriage may be facilitating widespread transmission in this population and is potentially a significant barrier to effective control since asymptomatic screening for BEPs is not recommended. Important knowledge gaps include the duration of infectiousness (pathogens with a longer duration of infectiousness can persist in the population) and the probability of transmission between an asymptomatic carrier and a susceptible person, which might better inform control measures. There are likely to be biological differences between individual BEPs that influence the probability of the pathogen being transmitted through sexual contact in MSM.

The higher prevalence of *mphA* detected among men with a BEP is consistent with the gene often being present in these pathogens, although it is likely that *mphA* was also present in other gut organisms. Our findings emphasise that frequent antimicrobial use in this population has important potential consequences for the development of resistance in both pathogenic and non-pathogenic gut microbes. The latter might act as a reservoir for AMR genes that can be transferred to other pathogens.^12^ Since 2018, STI treatment guidelines have shifted away from the use of azithromycin due to the development and spread of resistance in target and non-target pathogens.^22^ For instance, gonorrhoea treatment guidelines were updated in early 2019 to single dose ceftriaxone therapy from the previously recommended dual therapy of ceftriaxone and azithromycin.^29^ However, the recent rise in cases of extremely drug resistant *Shigella sonnei* (including genes conferring resistance to ceftriaxone) among MSM in the UK and Europe continues to emphasise the importance of antimicrobial stewardship and appropriate clinical management.^30^^,^^31^ There are also concerns about the potential overuse of antimicrobials in this population as prophylaxis for bacterial STIs. Although not currently recommended, studies have reported that up to 10% of MSM taking PrEP have used antibiotics to prevent STIs,^32^, ^33^, ^34^ primarily doxycycline, but the use of other antibiotics, including azithromycin, has also been reported.^35^ MSM reporting STI-risk behaviours acquire more STIs and BEPs and have greater exposure to antimicrobials. There is clearly a need to review current guidelines on antimicrobial use in MSM; the parallel syndemics of increasingly resistant sexually transmissible pathogens need to be addressed holistically.

## Contributors

HDM, GH and NF designed the study with input from GW, CJ and NRT. HDM, GH and NF developed the study protocol, and secured ethics and other regulatory approvals. HDM coordinated and managed the implementation of the study and the collection of swabs, with support from GW. GW, JZ and JO undertook the extraction of data from the clinic database. MB and PBB extracted GUMCAD data and performed data linkage. HDM processed the rectal swabs and performed the laboratory procedures, overseen by CJ and PS. HDM undertook the data analyses and wrote the first draft of the manuscript. All authors read, commented on, and approved the final version of the manuscript submitted for publication.

## Conference presentations

STI & HIV 2019 World Congress - Joint Meeting of the 23rd International Society for Sexually Transmitted Diseases Research (ISSTDR) & 20th International Union against Sexually Transmitted Infections (IUSTI), 14 to 17 July 2019, Vancouver, Canada.

## Data sharing statement

The individual participant data that underlie the results reported in this article cannot be shared due to the opt-out nature of the study design. The study utilises data from the GUMCAD STI Surveillance System which is managed by the UK Health Security Agency (formerly Public Health England). These data cannot be shared publicly, and their storage and access are under strict control. For the purposes of Open Access, the authors have applied a CC BY public copyright licence to any Author Accepted Manuscript version arising from this submission.

## Funding statement

This research was funded through the National Institute for Health Research Health Protection Research Unit (NIHR HPRU) in Blood Borne and Sexually Transmitted Infections at University College London in partnership with the UK Health Security Agency (UKHSA, formerly Public Health England), in collaboration with the London School of Hygiene and Tropical Medicine, and the NIHR HPRU in Gastrointestinal Infections at the University of Liverpool in partnership with UKHSA, in collaboration with the University of East Anglia, the University of Oxford and the Quadram Institute. HDM, MB, PBB and GH are affiliated to the NIHR HPRU in Blood Borne and Sexually Transmitted Infections. CJ is affiliated to the NIHR HPRU in Gastrointestinal Infections. The views expressed are those of the authors and not necessarily those of the NIHR, the Department of Health and Social Care or UKHSA. NRT was funded in whole by the 10.13039/100010269Wellcome Trust (grant 206194).

## Declaration of Competing Interest

The authors declare that there are no conflicts of interest.

## Appendix Supplementary materials

Image, application 1

Image, application 2

Image, application 3

Image, application 4

Image, application 5
