# Supplementary material for: Prevalence and risk factors of bacterial enteric pathogens in men who have sex with men: A cross-sectional study at the UK's largest sexual health service
Source: J Infect. 2023 Jan;86(1):33–40. doi: 10.1016/j.jinf.2022.10.033 (PMC10564623; doi:10.1016/j.jinf.2022.10.033)
Supplement: Supplementary file 1 [file mmc1.docx]

**Supplementary Data**

**Handling of missing data**

The distribution of missing clinical, socio-demographic and behavioural data was assessed to understand the mechanism of missing data and to guide the appropriate choice of methods for handling missing data.

Missing demographic data ranged from 0.4% (9/2116) for age group to 4.6% (97/2116) for region of birth. The percentage of missing behavioural data ranged from 9·8% (208/2116) for receptive anal sex in the past three months to 23·4% (494/2116) for number of new sexual partners in the past three months. Among people who had completed the clinical proforma, 23·7% (493/2082) did not provide any information on symptoms of gastroenteritis or diarrhoeal illness, and this was taken to indicate the absence of symptoms.

Key behavioural data (number of sexual partners, number of new sexual partners, last condomless sex, receptive anal sex, receptive oral sex and ‘interest in specific high-risk practices’) were complete for 74·7% (1581/2116) of study participants. However, there were systematic differences between people with observed and missing data. For instance, people with missing data for all these variables were more likely to have been diagnosed with gonorrhoea in the past 42 days (24.4% vs 3.5%, p<0.001), to have attended 56 Dean Street (56DS) (56.3% vs 16.7%, p<0.001), to be living with HIV (52.6% vs 15.2%, p<0.001), and to be of an ethnic minority group (30.8% vs 21.6%, p=0.015) compared to those with at least one variable completed. The pattern of missing data varied according to the clinic attended and was likely related to the division in service provision across the two clinics. Among 56DS attendees, people with missing data for all variables were more likely to be living with HIV (77.6 vs 19.6%, p<0.001). However, there was no evidence for an association between HIV status and missing data among people attending Dean Street Express (DSE) (20.3% all missing data vs 14.3% with at least one complete variable, p=0.196). This difference may be related to the fact that people living with HIV attend 56DS specifically for HIV care, and a clinical proforma may not be routinely completed during this type of consultation. STI swabs may be taken opportunistically at these visits either without or with only partial completion of the clinical proforma. At both clinics, there was a strong association between a recent (past 42 days) diagnosis of gonorrhoea and missing behavioural data, suggesting that some people may have been attending for test-of-cure swabs where a clinical proforma was not routinely completed (DSE: 30.0% vs 3.7%, p<0.001, 56DS: 15.7% vs 2.5%, p<0.001). When restricted to people who had information for at least one behavioural variable, people with missing data for at least one data item were more likely to have attended 56DS compared to those with complete data. Among people attending DSE, older age and HIV-negative/unknown status were associated with missing data.

These analyses suggested that there were multiple reasons for missing data. Clinical proforma completion was likely related to the reason for attendance as well as the clinic attended. People taking HIV pre-exposure prophylaxis (PrEP) usually complete a PrEP specific clinical proforma. Missing PrEP status was therefore dependent on whether the individual was taking PrEP and those who were taking PrEP were more likely to have missing data. These analyses suggested that the missing data mechanism in the study may be missing not at random.

Sensitivity analyses using simple imputation methods were conducted to assess the potential bias of missing data on the results. First, the missing indicator method was applied whereby missing data were grouped into an additional category, thus the full dataset was retained. Second, sensitivity analyses using worst-case and best-case scenarios were performed for all behavioural factors of interest and symptom data, where missing data were systematically replaced with the lowest or highest observed values to test the effect. Finally, single value imputation was used to assess the association between bacterial enteric pathogen (BEP) detection and partner number by replacing missing values for the number of sexual partners with the median value. In this final sensitivity analysis, different categories of partner number were explored in addition to the inclusion of partner number as a continuous variable.

**Sensitivity analyses: primary results**

Sensitivity analyses using simple imputation methods supported the findings presented in the primary results and strengthen their validity:

- Using the missing indicator method, the strength of association between BEP detection and each behavioural variable was similar to the primary analyses.
- Replacing missing behavioural data with the lowest observed value attenuated the measures of association for most variables, but the same factors remained associated with a prevalent BEP. On the other hand, in this model, the strength of association was accentuated for ‘interest in specific high-risk practices’ and last condomless sex.
- Replacing missing values with the highest observed value for each behavioural variable somewhat attenuated the strength of association in the highest category. However, the same factors remained associated with the detection of a BEP.
- Higher partner number was strongly associated with the detection of a BEP after replacing missing values with the median value. The associations remained after adjusting for age group, clinic and HIV status.

**Sensitivity analyses: stratification by HIV status**

Sensitivity analyses were conducted to assess the impact of missing data on the findings. Among men who were HIV-negative or of unknown HIV status, the results from the sensitivity analyses were similar to those from the main analysis:

- Using the missing indicator method, the findings supported those presented in the main analyses and the direction of the association was similar.
- Replacing missing behavioural data with the lowest observed value resulted in some or no attenuation to the prevalence ratio, and the same factors remained associated with BEP detection. Compared to the main analysis, there was stronger evidence to suggest that recent condomless sex was associated with BEP detection and the prevalence ratios were slightly accentuated.
- Replacing missing behavioural data with the highest observed value attenuated the prevalence ratio for the highest category. Compared to the main analysis, the same variables remained associated with BEP detection, except for last condomless sex where there was no evidence for an association with BEP detection.
- Higher partner number was strongly associated with the detection of a BEP after replacing missing values with the median value.

Among men living with HIV, the results from the sensitivity analyses were mixed and for some variables, there were conflicting findings:

- Using the missing indicator method, men who reported an ‘interest in specific high-risk practices’ were less likely to have a BEP detected, as reported in the primary analyses. However, there was weaker evidence to suggest that men who engaged in receptive oral sex in the past three months were less likely to have a BEP detected, after adjusting for age group and clinic (adjusted prevalence ratio: 0.32 [95% CI: 0.10 to 1.04], p=0.068). There was no evidence for any difference in the prevalence of a BEP according to reported symptoms.
- After replacing missing values with the lowest observed value, there was little evidence to suggest that men who reported an ‘interest in specific high-risk practices’ were less likely to have a BEP detected (adjusted prevalence ratio: 0.57 [95% CI: 0.29 to 1.11], p=0.098). Men who had symptoms of gastroenteritis were more likely to have a BEP detected, after adjusting for age group and clinic (adjusted prevalence ratio: 4.15 [95% CI: 1.08-16.0], p=0.038).
- After replacing missing values with the highest observed value, there was strong evidence to suggest that men who reported an ‘interest in specific high-risk practices’ were less likely to have a BEP detected (adjusted prevalence ratio: 0.45 [95% CI: 0.26-0.81], p=0.007). Men who reported receptive oral sex in the past three months were less likely to have a BEP detected, although this was of borderline significance after adjusting for age group and clinic (adjusted prevalence ratio: 0.30 [95% CI: 0.09 to 1.03], p=0.055). There was no evidence for any difference in the prevalence of a BEP according to reported symptoms of gastroenteritis.
- There was no evidence for an association between partner number and the detection of a BEP after replacing the missing values with the median number of sexual partners.
