# Supplementary material for: Prevalence and risk factors of bacterial enteric pathogens in men who have sex with men: A cross-sectional study at the UK's largest sexual health service
Source: J Infect. 2023 Jan;86(1):33–40. doi: 10.1016/j.jinf.2022.10.033 (PMC10564623; doi:10.1016/j.jinf.2022.10.033)
Supplement: Supplementary file 2 [file mmc2.docx]

**Supplementary Table 1: Primers, probes and gene targets for polymerase chain reaction (PCR) assays**

| Causative agent | Target | Name | Sequence (5’ – 3’) | 5’ mod | 3’ mod | Gene targeted |
| --- | --- | --- | --- | --- | --- | --- |
| *Campylobacter jejuni* | mapA | mapA Forward | CTGGTGGTTTTGAAGCAAAGATT |  |  | Gene specific for *C. Jejuni* (mapA) |
|  | mapA | mapA Reverse | CAATACCAGTGTCTAAAGTGCGTTTAT |  |  |  |
|  | mapA | mapA Probe | TTGAATTCCAACATCGCTAATGTATAAAAGCCCTTT | FAM | BHQ1 |  |
| *Campylobacter coli* | ceuE | ceuC Forward | AAGCTCTTATTGTTCTAACCAATTCTAACA |  |  | Gene specific for *C. Coli* (ceuE) |
|  | ceuE | ceuC Reverse | TCATCCACAGCATTGATTCCTAA |  |  |  |
|  | ceuE | ceuC Probe | TTGGACCTCAATCTCGCTTTGGAATCATT | Vic/YY | BHQ2 |  |
| *Salmonella* spp. | Ttr6 | ttr Forward | CTCACCAGGAGATTACAACATGG |  |  | ttr |
|  | Ttr4 | ttr Reverse | AGCTCAGACCAAAAGTGACCATC |  |  |  |
|  | Ttr5 | ttr Probe | CACCGACGGCGAGACCGACTTT | FAM | BHQ1 |  |
| STEC | stx1 | stx1 Forward | GGATAATTTGTTTGCAGTTGATGTC |  |  | stx1 |
|  | stx1 | stx1 Reverse | CAAATCCTGTCACATATAAATTATTTCGT |  |  |  |
|  | stx1 | stx1 Probe | CGTAGATTATTAAACCGCCCTCCTCTGGA | Cy5 | BHQ2 |  |
|  | stx2 | stx2 Forward | TTTGTYACTGTSACAGCWGAAGCYTTACG |  |  | stx2 |
|  | stx2 | stx2 Reverse | CCCCAGTTCARWGTRAGRTCMACRTC |  |  |  |
|  | stx2 | stx2 Probe | TCGTCAGGCACTGTCTGAAACTGCTCC | YY | BHQ2 |  |
| EPEC | eae | eae Forward | CATTGATCAGGATTTTTCTGGTGATA |  |  | eae |
|  | eae | eae Reverse | CTCATGCGGAAATAGCCGTTA |  |  |  |
|  | eae | eae Probe | ATAGTCTCGCCAGTATTCGCCACCAATACC | JOE | BHQ1 |  |
| EAEC | aagR | aagR Forward | CCATTTATCGCAATCAGATTAA |  |  | aggR |
|  | aagR | aagR Reverse | CAAGCATCTACTTTTGATATTCC |  |  |  |
|  | aagR | aagR Probe | CAGCGATACATTAAGACGCCTAAAGGA | Cy5 | BHQ2 |  |
| *Shigella* spp./Enteroinvasive *E. coli* | ipaH | ipaH Forward | AGGTCGCTGCATGGCTGGAA |  |  | ipaH |
|  | ipaH | ipaH Reverse | CACGGTCCTCACAGCTCTCA |  |  |  |
|  | ipaH | ipaH Probe | AACTCAGTGCCTCTGCGGAGCTTCGACA | FAM | BHQ1 |  |
| Green fluorescent protein *E. coli* | gfp | gfp Forward | CCTGTCCTTTTACCAGACAACCA |  |  | gfp |
|  | gfp | gfp Reverse | GGTCTCTCTTTTCGTTGGGATCT |  |  |  |
|  | gfp | gfp Probe | TACCTGTCCACACAATCTGCCCTTTCG | Cy5 | BHQ2 |  |

Abbreviations: STEC, Shiga toxin-producing *E. coli*; EAEC; EPEC, Enteropathogenic *E. coli*; Enteroaggregative *E. coli*; Gpf, Green fluorescent protein
