# Supplementary material for: Prevalence and risk factors of bacterial enteric pathogens in men who have sex with men: A cross-sectional study at the UK's largest sexual health service
Source: J Infect. 2023 Jan;86(1):33–40. doi: 10.1016/j.jinf.2022.10.033 (PMC10564623; doi:10.1016/j.jinf.2022.10.033)
Supplement: Supplementary file 3 [file mmc3.docx]

**Supplementary Table 2: Comparison of socio-demographic characteristics of the study population with all men who have sex with men attending sexual health clinics during the study period**

| Characteristic |  | MSM 16+ years attending clinics during the study period^a^ | | |
| --- | --- | --- | --- | --- |
|  | Study population  n (%)  N=2,116 | 56 Dean Street n (%)  N=5,286 | London SHCs  n (%)  N=12,449 | England SHCs  n (%)  N=22,740 |
| Age group |  |  |  |  |
| 16-19 | 31 (1.5) | 51 (1.0) | 191 (1.5) | 759 (3.4) |
| 20-24 | 241 (11.4) | 608 (11.5) | 1,413 (11.4) | 3,416 (15.1) |
| 25-29 | 526 (25.0) | 1,291 (24.4) | 2,708 (21.8) | 4,820 (21.5) |
| 30-34 | 485 (23.0) | 1,193 (22.6) | 2,602 (20.9) | 4,150 (18.3) |
| 35-39 | 337 (16.0) | 859 (16.3) | 1,919 (15.4) | 2,995 (13.2) |
| 40-49 | 339 (16.1) | 887 (16.8) | 2,276 (18.3) | 3,720 (16.4) |
| 50+ | 148 (7.0) | 397 (7.5) | 1,335 (10.7) | 2,820 (12.4) |
| Missing | 9 | 0 | 5 | 60 |
| Ethnic group |  |  |  |  |
| White | 1,576 (77.8) | 4,032 (79.0) | 8,672 (74.8) | 17,169 (80.9) |
| Black | 76 (3.8) | 218 (4.3) | 776 (6.7) | 976 (4.6) |
| Mixed | 131 (6.5) | 288 (5.6) | 662 (5.7) | 970 (4.6) |
| Asian | 112 (5.5) | 252 (4.9) | 728 (6.3) | 1,134 (5.3) |
| Other | 130 (6.4) | 313 (6.1) | 751 (6.5) | 974 (4.6) |
| Missing | 91 | 183 | 860 | 1,517 |
| World region of Birth |  |  |  |  |
| UK | 953 (47.2) | 2,554 (50.4) | 5,809 (51.4) | 13,817 (66.3) |
| Europe | 581 (28.8) | 1,374 (27.1) | 2,886 (25.5) | 3,657 (17.6) |
| Asia | 158 (7.8) | 362 (7.1) | 873 (7.7) | 1,246 (6.0) |
| South America | 122 (6.0) | 253 (5.0) | 615 (5.4) | 694 (3.3) |
| North America | 53 (2.6) | 158 (3.1) | 162 (1.4) | 209 (1.0) |
| Central America & the Caribbean | 24 (1.2) | 56 (1.1) | 273 (2.4) | 319 (1.5) |
| Africa | 69 (3.4) | 156 (3.1) | 386 (3.4) | 556 (2.7) |
| Australasia | 59 (3.0) | 159 (3.1) | 303 (2.7) | 342 (1.6) |
| Missing | 97 | 214 | 1,142 | 1,900 |
| Patient residence |  |  |  |  |
| London | 1,928 (92.3) | 4,782 (90.5) | 10,880 (92.6) | 10,954 (50.1) |
| UK elsewhere | 162 (7.8) | 461 (8.7) | 874 (7.4) | 10,905 (49.9) |
| Missing | 26 | 43 | 695 | 881 |

^a^ MSM clinic attendees who had a sample collected for chlamydia and gonorrhoea testing as reported through the GUMCAD STI Surveillance System. Abbreviations: MSM, Men who have sex with men, SHCs; Sexual Health Clinic
