# Supplementary material for: Prevalence and risk factors of bacterial enteric pathogens in men who have sex with men: A cross-sectional study at the UK's largest sexual health service
Source: J Infect. 2023 Jan;86(1):33–40. doi: 10.1016/j.jinf.2022.10.033 (PMC10564623; doi:10.1016/j.jinf.2022.10.033)
Supplement: Supplementary file 4 [file mmc4.docx]

**Supplementary Table 3: Associations of socio-demographic, clinical and behavioural factors with the detection of any bacterial enteric pathogen in HIV-negative/unknown status men who have sex with men**

| Factor | n/N | Row % | Unadjusted PR  (95% CI) | p-value | Adjusted PR  (95% CI) | p-value |
| --- | --- | --- | --- | --- | --- | --- |
| Clinic (N=1744) |  |  |  |  |  |  |
| DSE | 140/1461 | 9.6 | 1.00 | 0.445 | 1.00 | 0.456 |
| 56DS | 23/283 | 8.1 | 0.85 (0.56-1.29) |  | 0.85 (0.56-1.30) |  |
| Age group (N=1736) |  |  |  |  |  |  |
| 16-24 | 17/262 | 6.5 | 1.00 | 0.129 | 1.00 | 0.131 |
| 25-34 | 80/868 | 9.2 | 1.42 (0.86-2.35) | 0.043^a^ | 1.41 (0.85-2.34) | 0.044^a^ |
| 35+ | 66/606 | 10.9 | 1.68 (1.00-2.80) |  | 1.67 (1.00-2.80) |  |
| Ethnic group (N=1667) |  |  |  |  |  |  |
| White | 136/1304 | 10.4 | 1.00 | 0.149 | 1.00 | 0.185 |
| Black | 5/56 | 8.9 | 0.86 (0.37-2.01) |  | 0.87 (0.37-2.02) |  |
| Mixed | 3/108 | 2.8 | 0.27 (0.09-0.82) |  | 0.28 (0.09-0.87) |  |
| Asian | 8/96 | 8.3 | 0.80 (0.40-1.58) |  | 0.85 (0.43-1.68) |  |
| Other | 7/103 | 6.8 | 0.65 (0.31-1.36) |  | 0.66 (0.32-1.36) |  |
| Region of birth (N=1664) |  |  |  |  |  |  |
| UK | 71/802 | 8.9 | 1.00 | 0.549 | 1.00 | 0.577 |
| Europe | 51/477 | 10.7 | 1.21 (0.86-1.70) |  | 1.20 (0.85-1.69) |  |
| Rest of world | 38/385 | 9.9 | 1.11 (0.77-1.62) |  | 1.09 (0.75-1.58) |  |
| IMD quintile (N=1714) |  |  |  |  |  |  |
| 1-2 (Most deprived) | 108/1149 | 9.4 | 1.00 | 0.990 | 1.00 | 0.979 |
| 3 | 29/313 | 9.3 | 0.99 (0.67-1.46) |  | 0.98 (0.66-1.44) |  |
| 4-5 (Least deprived) | 23/252 | 9.1 | 0.97 (0.63-1.49) |  | 0.96 (0.62-1.48) |  |
| Sexual orientation (N=1717) |  |  |  |  |  |  |
| Gay | 157/1641 | 9.6 | 1.00 | 0.394 | 1.00 | 0.416 |
| Bisexual/heterosexual | 5/76 | 6.6 | 0.69 (0.29-1.63) |  | 0.70 (0.30-1.66) |  |
| HIV PrEP use (N=1477) |  |  |  |  |  |  |
| No | 60/930 | 6.5 | 1.00 | <0.001 | 1.00 | <0.001 |
| Yes | 74/547 | 13.5 | 2.10 (1.52-2.90) |  | 2.04 (1.47-2.84) |  |
| Bacterial STI diagnosed at attendance (N=1744) |  |  |  |  |  |  |
| No/unknown | 116/1357 | 8.6 | 1.00 | 0.031 | 1.00 | 0.028 |
| Yes | 47/387 | 12.1 | 1.42 (1.03-1.96) |  | 1.43 (1.04-1.97) |  |
| Bacterial STI diagnosed in previous year (N=1744) |  |  |  |  |  |  |
| No/unknown | 85/1078 | 7.9 | 1.00 | 0.008 | 1.00 | 0.010 |
| Yes | 78/666 | 11.7 | 1.49 (1.11-1.99) |  | 1.47 (1.10-1.97) |  |
| Interest in specific high-risk practices^b^ (N=1501) |  |  |  |  |  |  |
| No | 72/941 | 7.7 | 1.00 | 0.001 | 1.00 | 0.002 |
| Yes | 72/560 | 12.9 | 1.68 (1.23-2.29) |  | 1.63 (1.19-2.23) |  |
| Number of sexual partners in last 3 months (N=1416) |  |  |  |  |  |  |
| 0-1 | 12/148 | 8.1 | 1.00 | 0.003 | 1.00 | 0.005 |
| 2-4 | 35/570 | 6.1 | 0.76 (0.40-1.42) | <0.001^a^ | 0.72 (0.39-1.35) | 0.003^a^ |
| 5-9 | 41/370 | 11.1 | 1.37 (0.74-2.53) |  | 1.28 (0.70-2.36) |  |
| 10+ | 44/328 | 13.4 | 1.65 (0.90-3.04) |  | 1.54 (0.83-2.81) |  |
| Number of new sexual partners in last 3 months (N=1351) |  |  |  |  |  |  |
| 0-1 | 18/355 | 5.1 | 1.00 | <0.001 | 1.00 | <0.001 |
| 2-4 | 41/505 | 8.1 | 1.60 (0.94-2.74) | <0.001^a^ | 1.59 (0.94-2.70) | <0.001^a^ |
| 5-9 | 38/286 | 13.3 | 2.62 (1.53-4.49) |  | 2.58 (1.51-4.39) |  |
| 10+ | 32/205 | 15.6 | 3.08 (1.77-5.34) |  | 2.97 (1.71-5.13) |  |
| Receptive anal sex in last 3 months (N=1622) |  |  |  |  |  |  |
| No | 5/85 | 5.9 | 1.00 | 0.299 | 1.00 | 0.300 |
| Yes | 143/1537 | 9.3 | 1.58 (0.67-3.76) |  | 1.57 (0.67-3.67) |  |
| Receptive oral sex in last 3 months (N=1590) |  |  |  |  |  |  |
| No | 4/42 | 9.5 | 1.00 | 0.926 | 1.00 | 0.868 |
| Yes | 141/1548 | 9.1 | 0.96 (0.37-2.46) |  | 0.92 (0.36-2.37) |  |
| Last condomless sex (N=1605) |  |  |  |  |  |  |
| Never or more than 6 weeks ago | 49/566 | 8.7 | 1.00 | 0.045 | 1.00 | 0.053 |
| Within 6 weeks | 73/811 | 9.0 | 1.04 (0.74-1.47) |  | 1.03 (0.73-1.46) |  |
| Within 72 hours | 32/228 | 14.0 | 1.62 (1.07-2.46) |  | 1.60 (1.05-2.43) |  |
| Gastrointestinal symptoms (N=1715) |  |  |  |  |  |  |
| No/unknown | 159/1686 | 9.4 | 1.00 | 0.867 | 1.00 | 0.662 |
| Yes | 3/29 | 10.3 | 1.10 (0.37-3.24) |  | 1.28 (0.42-3.93) |  |

Total numbers vary for each question due to missing data. Unadjusted and adjusted prevalence ratios (PRs) and 95% confidence intervals (CIs) calculated using modified Poisson regression with robust error variance. Overall p-values by Wald test or linear test for trend (^a^). Adjusted Models: Each factor adjusted in separate model for age group (linear term) and clinic. ^b^‘Interest in specific high-risk practices’ refers to data collected via the following question: Are you into any of these: Fisting, injecting, bare backing, chemsex. Abbreviations: IMD, Index of Multiple Deprivation; STI, Sexually Transmitted Infection; PrEP, Pre-exposure prophylaxis.
