# Supplementary material for: Prevalence and risk factors of bacterial enteric pathogens in men who have sex with men: A cross-sectional study at the UK's largest sexual health service
Source: J Infect. 2023 Jan;86(1):33–40. doi: 10.1016/j.jinf.2022.10.033 (PMC10564623; doi:10.1016/j.jinf.2022.10.033)
Supplement: Supplementary file 5 [file mmc5.docx]

**Supplementary Table 4: Associations of socio-demographic, clinical and behavioural factors with the detection of any bacterial enteric pathogen in men who have sex with men living with HIV**

| Factor | n/N | Row % | Unadjusted  PR (95% CI) | p-value | Adjusted  PR (95% CI) | p-value |
| --- | --- | --- | --- | --- | --- | --- |
| Clinic (N=372) |  |  |  |  |  |  |
| DSE | 34/248 | 13.7 | 1.00 | 0.122 | 1.00 | 0.143 |
| 56DS | 10/124 | 8.1 | 0.59 (0.30-1.15) |  | 0.60 (0.30-1.19) |  |
| Age group (N=371) |  |  |  |  |  |  |
| 16-34 | 20/153 | 13.1 | 1.00 | 0.546 | 1.00 | 0.609 |
| 35+ | 24/218 | 11.0 | 0.84 (0.48-1.47) |  | 0.86 (0.49-1.52) |  |
| Ethnic group (N=358) |  |  |  |  |  |  |
| White | 33/272 | 12.1 | 1.00 | 0.901 | 1.00 | 0.906 |
| Ethnic minority | 10/86 | 11.6 | 0.96 (0.49-1.86) |  | 0.96 (0.50-1.84) |  |
| Region of birth (N=355) |  |  |  |  |  |  |
| UK | 19/151 | 12.6 | 1.00 | 0.865 | 1.00 | 0.885 |
| Europe | 14/104 | 13.5 | 1.07 (0.56-2.04) |  | 1.01 (0.53-1.95) |  |
| Rest of world | 11/100 | 11.0 | 0.87 (0.43-1.76) |  | 0.86 (0.43-1.71) |  |
| IMD quintile (N=371) |  |  |  |  |  |  |
| 1-2 (Most deprived) | 36/258 | 14.0 | 1.00 | 0.192 | 1.00 | 0.197 |
| 3 | 4/61 | 6.6 | 0.47 (0.17-1.27) |  | 0.47 (0.17-1.25) |  |
| 4-5 (Least deprived) | 4/52 | 7.7 | 0.55 (0.20-1.48) |  | 0.58 (0.22-1.55) |  |
| Sexual orientation (N=365) |  |  |  |  |  |  |
| Gay | 43/362 | 11.9 | NA |  |  |  |
| Bisexual/heterosexual | 0/3 | 0 |  |  |  |  |
| Bacterial STI diagnosed at attendance (N=372) |  |  |  |  |  |  |
| No/unknown | 29/275 | 10.6 | 1.00 | 0.195 | 1.00 | 0.208 |
| Yes | 15/97 | 15.5 | 1.47 (0.82-2.62) |  | 1.45 (0.81-2.56) |  |
| Bacterial STI diagnosed in last year (N=372) |  |  |  |  |  |  |
| No/unknown | 18/173 | 10.4 | 1.00 | 0.431 | 1.00 | 0.679 |
| Yes | 26/199 | 13.1 | 1.26 (0.71-2.21) |  | 1.13 (0.63-2.04) |  |
| Interest in specific high-risk practices^a^ (N=274) |  |  |  |  |  |  |
| No | 25/133 | 18.8 | 1.00 | 0.026 | 1.00 | 0.029 |
| Yes | 13/141 | 9.2 | 0.49 (0.26-0.92) |  | 0.49 (0.26-0.93) |  |
| Number of sexual partners in last 3 months (N=286) |  |  |  |  |  |  |
| 0-4 | 19/142 | 13.4 | 1.00 | 0.963 | 1.00 | 0.973 |
| 5+ | 19/144 | 13.2 | 0.99 (0.54-1.78) |  | 0.99 (0.54-1.81) |  |
| Number of new sexual partners in last 3 months (N=271) |  |  |  |  |  |  |
| 0-4 | 23/170 | 13.5 | 1.00 | 0.878 | 1.00 | 0.887 |
| 5+ | 13/101 | 12.9 | 0.95 (0.50-1.80) |  | 0.96 (0.50-1.82) |  |
| Receptive anal sex in last 3 months (N=286) |  |  |  |  |  |  |
| No | 2/7 | 28.6 | 1.00 | 0.214 | 1.00 | 0.180 |
| Yes | 37/279 | 13.3 | 0.46 (0.14-1.56) |  | 0.42 (0.12-1.49) |  |
| Receptive oral sex in last 3 months (N=284) |  |  |  |  |  |  |
| No | 2/5 | 40.0 | 1.00 | 0.053 | 1.00 | 0.053 |
| Yes | 37/279 | 13.3 | 0.33 (0.11-1.01) |  | 0.32 (0.10-1.01) |  |
| Last condomless sex (N=268) |  |  |  |  |  |  |
| Never/more than 6 weeks ago | 5/55 | 9.1 | 1.00 | 0.314 | 1.00 | 0.319 |
| Within 6 weeks | 32/222 | 14.4 | 1.59 (0.65-3.89) |  | 1.59 (0.64-3.94) |  |
| Gastrointestinal symptoms (N=367) |  |  |  |  |  |  |
| No/unknown | 42/360 | 11.7 | 1.00 | 0.146 | 1.00 | 0.040 |
| Yes | 2/7 | 28.6 | 2.45 (0.73-8.19) |  | 4.09 (1.07-15.7) |  |

Total numbers vary for each question due to missing data. Unadjusted and adjusted prevalence ratios (PRs) and 95% confidence intervals (CIs) calculated using modified Poisson regression with robust error variance. Overall p-values by Wald test. Adjusted Models: Each factor adjusted in separate model for age group (linear term) and clinic. ^a^‘Interest in specific high-risk practices’ refers to data collected via the following question: Are you into any of these: Fisting, injecting, bare backing, chemsex. Abbreviations: IMD, Index of Multiple Deprivation; STI, Sexually Transmitted Infection
